# Headache in Behcet’s disease: types and characteristics

**DOI:** 10.1186/s40064-016-2721-4

**Published:** 2016-07-13

**Authors:** Maryam Moghaddassi, Mansoureh Togha, Farhad Shahram, Hamed Hanif, Sahar Dadkhah, Soodeh Razeghi Jahromi, Mohammad Mozafari

**Affiliations:** Rheumatology Research Center, Tehran University of Medical Sciences, Tehran, Iran; Headache Department, Iranian Center of Neurological Research, Neuroscience Institute, Tehran University of Medical Sciences, Tehran, Iran; Department of Neurology, Neurology Ward, Sina Hospital, Tehran University of Medical Sciences, Hassan Abad Square, Tehran, 1136746911 Iran; Department of Neurosurgery, Birjand University of Medical Sciences, Birjand, Iran; Skin Research Center, Shahid Beheshti University of Medical Sciences, Tehran, Iran; Department of Clinical Nutrition and Dietetics, Faculty of Nutrition and Food Technology, Shahid-Beheshti University of Medical Sciences, Tehran, Iran

**Keywords:** Behcet’s disease, Headache, Migraine, Non-structural headache

## Abstract

**Background:**

Behcet’s disease involves several systems in the body. Neurological involvement is identified by different symptoms. Headache is one of the common complaints of patients with Behcet’s disease. It might be a part of neurological involvement or may arise independently in the course of disease. Studies with small sample size have resulted in various findings in this field. Since the prevalence of Behcet’s disease is relatively high in Iran, this study was carried out to compare the features of headache between an acceptable number of patients with this rare disease and a control group.

**Methods:**

The current case–control study was performed to compare the features of headache between 312 patients with definite Behcet’s disease who referred to a Behcet’s clinic and healthy individuals. Patients with Behcet’s disease were randomly selected. Controls were matched for age and sex. They were personally examined and interviewed meticulously using a questionnaire that met the standards of the International Headache Society classification for different types of headache.

**Results:**

The incidence of headache in the case and control groups was 28.3 % (n = 120) and 18.6 % (n = 59), respectively (p < 0.05; OR 2.73). Tension-type headache was observed in 12.2 % (n = 38) of cases which was significantly higher than control group (n = 6.3 %) (p = 0.011; OR 2.05). The most frequent type of headache in the case group was tension-type headache (12.2 %). In the control group, however, migraine without aura was the most common type (9.1 %). A correlation between ophthalmological involvement and headache was observed in 11 patients in the case group. In addition, a significantly higher systolic blood pressure was found in the case group compared to the controls (125.1 vs. 121.7 mmHg; p = 0.007). There was no major correlation between prednisolone consumption in patients with Behcet’s disease and the type and frequency of headache.

**Conclusions:**

Headache, especially tension-type headache, is more common in patients with Behcet’s disease. This might be the result of specific types of uveitis-related and non-structural headaches seen in Behcet’s disease.

## Background

Behcet’s disease (BD) is a multisystem, relatively rare disease that is considered to be one of the vasculitides (Sakane et al. 1999). BD is mostly seen in the countries alongside the Silk Road and particularly in the Middle East (Davatchi et al. 2008; Kaklamani et al. 1998; Kaneko et al. 2003; Mahr et al. 2008; Shahram et al. 2003; Cc 2003). BD is more prevalent in the male gender; according to a recent report from Iran, the male-to-female ratio was 3.1:1 (Davatchi et al. 2016). BD involves several systems in the body and the diagnosis is more of an expert opinion in the clinic. Although the clinical manifestations might vary individually, (Nakae et al. 1993; Tursen et al. 2003; Zhang et al. 2006), BD is diagnosed via the International Study Group Criteria in which definitive diagnosis necessitates recurrent oral ulcerations and two of the following: recurrent genital ulcers, distinctive skin lesions, eye lesions, and a positive pathergy test (Weichsler et al. 1990). In addition to these common features that confirm the diagnosis, BD contains minor manifestations like joint involvement, disorders of large vessels, and gastrointestinal, pulmonary, cardiac, and neurological syndromes. Among all neurological symptoms in BD, headache is the most frequent (Borhani Haghighi et al. 2005).

Headache occurrence in patients with BD might indicate onset of neuro-BD (NBD); however, the majority of these headaches are benign (Fountain and Dhurandhar 2014). Occasionally, BD is misdiagnosed as aseptic meningitis, multiple sclerosis, or a primary neoplasm (Fountain and Dhurandhar 2014). Although the prevalence of headache in BD is so high that some studies do not consider it as a sign for neurological involvement, it remains as a major complaint of many patients (Weichsler et al. 1990; Borhani Haghighi et al. 2005). In fact, the rate of neurological involvement varies from 2.5 to 49 % in different studies since some of them do not consider headache as a neurological symptom of BD (Weichsler et al. 1990; Borhani Haghighi et al. 2005). There are many untested theories concerning higher prevalence of specific types of headache in patients with BD (Davatchi et al. 2010; Monastero et al. 2003). For instance, a study in Iran illustrated that 57 % of patients experience tension-type headaches while 28.5 % complain from migraine. This study also mentioned an association between headaches and attacks of oral ulcers in about 12 % and uveoretinitis in 1.5 % of BD patients. However, it did not point out any significant difference between the cases and general population (Sikaroodi et al. 1992). Another questionnaire-based British study revealed the prevalence of recurrent headache to be 82.5 % in BD, with the dominant features of migraine headache pertaining to most headaches (Kidd 2003). Moreover, a separate prevalence study demonstrated a significantly higher frequency of migraine without aura in patients with BD (Monastero et al. 2003).

Specific types of headache which cannot be classified as primary and secondary and emerge in the course of BD are called “*non*-*structural headaches of BD*”. The prevalence of these types of headaches in BD shows notable variation among different studies (Sakane et al. 1999).

Many of the previous studies in this field have focused on the characteristics and specific treatments of headache in BD disease and all have reached challenging results (Monastero et al. 2003; Sikaroodi et al. 1992; Kidd 2003). Considering the disparities seen between previous investigations and high prevalence of BD in Iran, this study was designed to further delve into the frequency and characteristics of headache in BD with a larger number of cases.

## Methods

The current study was a case–control study performed at the multidisciplinary BD outpatient clinic. The total sample size was 629 (cases plus controls). For almost 4 months, all of the patients admitted to the BD outpatient clinic were consecutively included in the case group. In order to match the case and control groups in terms of socioeconomic characteristics, individuals in the control group were selected from patients in urology and orthopedic clinics of another hospital over a period of almost 5 months and matched for age and sex.

### Case group

The inclusion criterion was a definite diagnosis of BD and patients who did not agree to take part in this study were excluded. The diagnosis of BD was certain in patients in the case group, according to the BD classification criteria of the International Study Group. The patients were randomly selected and interviewed regardless of how long they have been diagnosed with BD. Each patient was individually interviewed and examined. A 2-part questionnaire was used for interviews. The first part included demographic information and general settings of the disease focusing on the clinical course of BD. The second part includes the headache characteristics according to the International Classification of Headache Disorders, second edition (ICHD-2) (2004) and the time relations between headache occurrence and first symptoms of BD. A trained general physician interviewed the patients and filled the questionnaire.

Primary headaches were classified as migraine with aura, migraine without aura, tension-type headaches, and cluster headaches. In accordance with a Turkish study, headaches that started 6 months before the onset of BD or any other headaches occurring after the onset of the disease which did not meet the criteria of international headache society (IHS) for primary headaches were classified as non-structural headaches related to BD (Tursen et al. 2003). Any headache along with neurological symptoms (except for classic associating symptoms of primary headaches) according to IHCD-2 was considered as NBD. After being confirmed by an ophthalmologist, severe localized headaches occurring during uveitis attacks were regarded as uveitis-related headaches.

For patients obtaining the characteristics of more than one specific type of headache, the most severe and classified one according to the criteria was investigated. For example, when a combination of a headache secondary to neurological involvement and any other type of headache was present, the first one was considered as the main headache. Patients who had any other clinical findings that could be related to other types of secondary headaches were referred for further investigation. When the proper diagnosis was made, the headaches were classified as one of the secondary types of headache such as headaches due to infections and sinusitis. Patients with reliable medical documents confirming their underlying disease that caused headaches were not sent for further evaluation.

### Control group

Subjects in the control group were selected from patients, without BD, who were based in urology and orthopedic clinics in another hospital. The control group included age- and sex-matched subjects for individuals in the case group. Each person filled the aforementioned questionnaire except for the part referring to the duration and onset of BD. Individuals that had any head and neck injury or any other internal disease that could be the reason of their headaches were excluded.

### Data analysis

Statistical package for the social sciences (SPSS) software for windows version 19.0 (IBM Inc., New York, NY, USA) was used to carry out the statistical analyses. In this analysis, α = 0.05 and β = 0.1. Also, 11 % difference in headache prevalence between the two groups was considered. The sample size was calculated to be at least 297 in each group. We used student’s t test for comparison of quantitative data and Chi square test for comparison of qualitative data. Moreover, P values less than 0.05 were considered significant.

### Ethical considerations

Patients voluntarily participated in this study. Their participation did not interfere with their normal treatment procedure. All of the patients provided written informed consents. Also, the ethics committee of Tehran University of Medical Sciences approved this study.

## Results

This study was performed on 312 (139 females and 173 males) patients in the case group and on 317 (138 females and 179 males) age- and sex-matched controls (Table 1). Both groups were similar in terms of sex distribution (p = 0.7). The mean age of participants was 39 years in both groups. The mean duration of BD was 10.3 ± 7.63 years in the case group. The mean systolic blood pressure was significantly different between the case and control groups (125.1 vs. 121.7 mmHg; p = 0.007) (Table 1).

**Table 1:** Age, systolic blood pressure and sex of participants in case and control groups

	Mean		Medium		Minimum		Maximum		95 % CI		P value
	Case	Control	Case	Control	Case	Control	Case	Control	Case	Control	
Age (years)	39.63	39.12	39	39	15	15	74	73	38.33–40.93	37.81–40.43	0.953
Blood pressure (mmHg)	125.1	121.7	125	120	90	90	170	160	123.2–126.9	120.3–123.1	0.007

Case		Control	
Sex			
Male	Female	Male	Female
173	139	179	138

*CI:* confidence interval

Our study revealed that the disease-related prevalence of headache was higher among patients with BD. The total prevalence of different types of headache was 28.3 and 18.6 % in the case and control groups, respectively. Tension-type headache was the most common type of headache in the case group with the prevalence of 12.2 %. However, the most common type of headache among the control group was migraine without aura with the prevalence of 9.1 %. It is noteworthy that the frequency of tension-type headache was significantly higher in female patients with BD (p = 0.002). There was no report of cluster headache or other autonomic neuralgias in either case or control groups (Table 2).

**Table 2:** Frequency and types of headaches in case and control groups

	Case (n = 312)	Control (n = 317)	Odds ratio	95 % CI	P value
Overall frequency of headaches, n (%)	120 (28.3 %)	59 (18.6 %)	2.73	1.9–3.9	>0.01
Migraine with aura, n (%)	12 (3.8 %)	8 (2.5 %)	1.5	0.6–3.8	0.345
Migraine without aura, n (%)	35 (11.2 %)	29 (9.1 %)	1.2	0.7–2.01	0.391
Tension-type headache, n (%)	38 (12.2 %)	20 (6.3 %)	2.05	1.17–3.62	0.011

*CI:* confidence interval

While headaches secondary to ophthalmological involvement were found in 11 (3.5 %) patients with BD, 10 patients had anterior uveitis. As Table 3 shows, there was a significant association between headache presence and only one BD manifestation and that manifestation was anterior uveitis (p = 0.025). Moreover, 15 patients (4.8 % of patients with BD) were suffering from non-structural headaches related to BD. However, there was no statistically significant correlation between this type of headache and severity (p = 0.170), quality (p = 0.082), and clinical course of BD (p = 0.089). All of these patients had normal magnetic resonance imaging (MRI) results and thus, were not diagnosed with NBD.

**Table 3:** Manifestations of Behcet’s disease and correlations with headache

Behcet’s disease symptoms		Overall	Headache		P value
			+	−	
Anterior uveitis	+	152	89	63	*0.025*
	−	160	104	56	
Posterior uveitis	+	188	116	72	0.944
	−	124	77	47	
Retinal vasculitis	+	151	99	52	0.192
	−	161	94	67	
Macular edema	+	19	9	10	0.180
	−	293	184	109	
Decrease of visual acuity	+	45	24	21	0.203
	−	267	169	98	
Genital aphthous	+	175	104	71	0.318
	−	137	89	48	
Erythema nodosum	+	77	52	25	0.238
	−	235	141	94	
Pseudofolliculitis	+	124	83	41	0.134
	−	188	110	78	
Cellulitis	+	1	1	0	0.432
	−	311	192	119	
Vasculitis	+	2	2	0	0.265
	−	310	191	119	
Aneurysm	+	1	1	0	0.432
	−	311	192	119	
Arthritis	+	140	82	58	0.582
	−	171	110	61	
Superficial and deep phlebitis	+	17	15	2	0.451
	−	294	177	117	
Central nervous system involvement	+	16	11	5	0.553
	−	195	181	114	
Seizure	+	6	5	1	0.272
	−	305	187	118	
Meningoencephalitis	+	1	1	0	0.432
	−	311	192	119	
Involvements of other organs	+	10	4	6	0.148
	−	310	189	113	

P values with italic font means they are significant (P values less than 0.05 were considered significant)

Interestingly, 15 patients with BD who did not suffer from headaches at the time of the study and were consequently not considered as cases of headache in the current study were found to have had a history of neurological involvement in the course of their BD. To be more precise, seven patients had a history of appropriately treated cerebral venous sinus thrombosis, four other patients were identified to have a history of brain stem involvement, three others had brain parenchymal lesions and another patient developed Behcet’s meningoencephalitis, as the primary presentation of BD, which was fully treated and did not recur.

The onset of headache was found to be years after the onset of BD in 58 % of patients. On the contrary, 20 % of the patients recalled having headaches years before they were diagnosed with BD. Moreover, 22 % of the cases reported the onset of headaches to be simultaneous with or within 6 months of BD diagnosis. The association between the onset of BD and timing of headaches was not statistically significant (p = 0.069).

Consumption of medications was another issue of interest. According to the performed analyses, the prevalence of headaches was not significantly different between patients who received prednisolone at a dose of 7.5 mg/kg daily and those who did not consume prednisolone. On the other hand, although the prevalence of headache was about 2 times higher in patients receiving 7.5 mg/kg daily and more (32.5 vs. 67 %), this difference was not statistically significant (p = 0.10).

Some patients of both the case and control groups (about 15 %) received specific treatment for their headaches and the two groups were not significantly different in this regard (Table 4).

**Table 4:** Drugs used for headache in the case and control groups

	Frequency in case group, n (%)	Frequency in control group, n (%)	Odds ratio	95 % CI	P value
Specific treatment for the headache	18 (15.1 %)	9 (15.3 %)	0.99	0.4–2.3	0.980
OTC analgesics	58 (48.7 %)	43 (72.9 %)	0.35	0.18–0.69	0.002
No treatment	43 (36.1 %)	7 (11.9 %)	4.2	1.7–10.0	0.001

*OTC:* over the counter, *CI:* confidence interval

In addition, headache frequency was not significantly associated with human leukocyte antigen (HLA) B51, HLA B5, and positive pathergy test (p = 0.15, 0.57, and 0.99, respectively).

The number of individuals with a positive family history of headache, especially migraine and tension-type headache, was not significantly different between the case and control groups (p = 0.99). However, it was significantly higher in individuals complaining from headaches in each group (Table 5).

**Table 5:** Family history of headaches in the case and control groups

	Family history, n		Odds ratio	95 % CI	P value
	Positive	Negative			
Case					
Headache +	28	92	1.86	1.03–3.34	0.037
Headache −	27	165			
Control					
Headache +	18	41	2.54	1.32–4.88	0.004
Headache −	38	220			

*CI:* confidence interval

We also compared 150 normotensive BD patients to 191 controls who had normal systolic blood pressure. The rate of headache in these groups was 59 and 41 %, respectively (p = 0.001; 95 % confidence interval: 1.4–3.8; odds ratio = 2.3).

## Discussion

BD is a multi-organ disease and the clinical features are mainly of mucocutaneous and ophthalmological origins (Davatchi et al. 2010). The most frequent early manifestations of neurological involvement in BD are gait disturbance, dysarthria, and headache (Yoon et al. 2014). As in any other population, headache is one of the most common symptoms of patients with BD. However, the prevalence of recurrent headache in BD has been estimated to be 82.5 %, with the features of migraine headache being the most dominant. In fact, headache can be a sign of neurological involvement in BD or may arise independently in the course of BD (Ashjazadeh et al. 2003).

The characteristics of primary headaches such as migraine, tension-type and cluster headaches, secondary headaches like those following ophthalmological involvement and non-structural headaches related to BD have been a field of interest in many studies. Meanwhile, the rarity and specific geographical distribution of BD have resulted in studies with small sample size. In this current, large-scale, cross-sectional study we found the overall prevalence of headache to be higher in patients with BD. Although similar to a previous study in our center, (Sikaroodi et al. 1992) the total prevalence of 28.3 % observed in our study was in contrast with studies in the United Kingdom (Kidd 2003), Italy (Monastero et al. 2003) and Turkey (Turkish Headache Epidemiology Study Group 1998), which all reported rates above 60 %.

Our study revealed tension-type headaches to be significantly more frequent among patients with BD than the control group. Sikaroodi et al. (1992) suggested similar findings. The frequency of non-structural headaches was found to be one-third of that reported in the study carried out in Turkey (Turkish Headache Epidemiology Study Group 1998). Patients with this kind of headache had a normal MRI and neither did they meet the criteria of the IHS nor had they any other underlying conditions that could be related to the headache. Additionally, we found no significant correlation between the onset of these headaches and other systemic symptoms such as aphthous ulcers.

Previous studies reported a link between blood pressure and headache (Harandi et al. 2013; Fagernaes et al. 2015). In the current study, the mean systolic blood pressure was significantly higher in patients with BD. This might be due to consumption of prednisolone or atherogenic pathologies of BD. In order to make sure that the difference in blood pressure does not cause dissimilarity in prevalence of headache among the case and control groups, we compared 150 patients with BD to 191 controls who had normal systolic blood pressure. Despite the similarity in blood pressure, the prevalence of headache was higher in BD group.

The frequency of uveitis-related headaches was found to be 3.5 % in this study, which was in agreement with other studies (Monastero et al. 2003; Kidd 2003). However, uveitis was the only symptom that established a significantly positive correlation with the general frequency of headaches in patients with BD.

The hypothesis that prednisolone reduces the prevalence of headache in BD was not confirmed in our study. This might be a result of the difference between the generally prescribed doses of prednisolone for patients with BD and doses required for treatment of headache. Prednisolone is rarely ordered for patients with headaches that do not respond to any other treatment. The usual dosage is at least 30 mg/daily for 2 weeks. Therefore, it seems rational that the common doses of prednisolone used for treatment of BD (7.5–15 mg/daily) exert a weak effect on headaches.

In some of our cases, an association was found between history of neurological involvement of BD and headache. Headaches were completely resolved after the treatment of neurological involvement. Although there was no significant association between NBD and headache prevalence, 15 out of 16 patients with neurological involvement reported history of severe headaches before the initiation of treatment for neurological lesions. After inquiring about the history of headache at the time of the neurological involvement, the relationship was found to be significant (p = 0.0001), with an odds ratio of 1.23. These findings are consistent with previous studies considering headache as the most common symptom in NBD (Ashjazadeh et al. 2003).

While in the United Kingdom 15 % of patients with BD are referred to hospitals to receive related medicine for headaches, (Kidd 2003) the referral rate turned out to be 5 % in our study. However, other reports in the country (Iran) have estimated a rate of 15 %. Considering social facilities in Iran, good insurance coverage and different levels of referral, 15 % does not seem that remarkable.

Our study revealed that the rate of treatment by over the counter analgesics versus non-medical treatments for headaches in BD was significantly different between the case and control groups. This might be due to lack of compliance of BD patients with receiving additional medicine, ergo their higher rate of applying non-medical treatments.

To be noted, in this study we found no specific pattern for intensity, characteristics, or localization of headaches in BD patients. Perhaps larger multi-center trials in the future can reveal any possible relationships between BD severity and duration and its major and minor symptoms and the above-mentioned variables of tension-type headache and migraine and even outline potential causal/temporal correlations. This feat has been achieved for other pain and rheumatologic disorders such as fibromyalgia syndrome and temporomandibular pain disorder (Giamberardino et al. 2015; Dahan et al. 2015).

The strengths of the current study were the large randomly selected population and the use of validated diagnostic criteria for headaches. As a limitation, the study was conducted on patients in a referral center and this might have rendered a lower coverage compared to a multi-center study.

## Conclusions

The overall prevalence of headache, especially tension-type headache, tends to be higher in patients with BD. Although headaches are among the most common complaints of BD patients, they are usually not appropriately considered and managed by their responsible physician.

## Abbreviations

BD: Behcet’s disease
NBD: neuro-Behcet’s disease
ICHD-2: international classification of headache disorders, second edition
IHS: international headache society
SPSS: statistical package for the social sciences
MRI: magnetic resonance imaging
